# eSIP: A Novel Solution-Based Sectioned Image Property Approach for Microscope Calibration

**DOI:** 10.1371/journal.pone.0134980

**Published:** 2015-08-05

**Authors:** Malte Butzlaff, Arwed Weigel, Evgeni Ponimaskin, Andre Zeug

**Affiliations:** 1 Cellular Neurophysiology, Center of Physiology, Hannover Medical School, Hannover, Germany; 2 Carl Zeiss Microscopy GmbH, Kistlerhofstr. 75, München, Germany; University of California, Berkeley, UNITED STATES

## Abstract

Fluorescence confocal microscopy represents one of the central tools in modern sciences. Correspondingly, a growing amount of research relies on the development of novel microscopic methods. During the last decade numerous microscopic approaches were developed for the investigation of various scientific questions. Thereby, the former qualitative imaging methods became replaced by advanced quantitative methods to gain more and more information from a given sample. However, modern microscope systems being as complex as they are, require very precise and appropriate calibration routines, in particular when quantitative measurements should be compared over longer time scales or between different setups. Multispectral beads with sub-resolution size are often used to describe the point spread function and thus the optical properties of the microscope. More recently, a fluorescent layer was utilized to describe the axial profile for each pixel, which allows a spatially resolved characterization. However, fabrication of a thin fluorescent layer with matching refractive index is technically not solved yet. Therefore, we propose a novel type of calibration concept for sectioned image property (SIP) measurements which is based on fluorescent solution and makes the calibration concept available for a broader number of users. Compared to the previous approach, additional information can be obtained by application of this extended SIP chart approach, including penetration depth, detected number of photons, and illumination profile shape. Furthermore, due to the fit of the complete profile, our method is less susceptible to noise. Generally, the extended SIP approach represents a simple and highly reproducible method, allowing setup independent calibration and alignment procedures, which is mandatory for advanced quantitative microscopy.

## Introduction

The field of sectioning fluorescence microscopy has rapidly advanced in recent years, offering experimenters to extract more and more information from a given sample by utilizing spectral analysis in multispectral systems, high temporal resolution in fast imaging systems, and high spatial resolution in systems employing molecular switches or structured illumination [1]. To yield quantitative information, all these systems have to be calibrated against a given standard. Routinely, multispectral μm-sized beads are used for alignment, colour calibration, and detection of intensity variations. Submicron-beads can be applied for measuring point-spread-functions (PSFs) to determine the systems’ resolution [2]. Theer *et al*. provided a promising application to extract in addition spatial information from bead samples [3]. In brightfield-microscopy, a common standard is to correct the images by a background subtraction, which smoothly removes specks from dirt in system compartments. In fluorescence microscopy, however, the awareness slowly arises that a comparable procedure is required for quantitative imaging, so standards are being developed accordingly [4, 5]. For example, an axial image stack of a homogeneous fluorescent layer yields information about intensity and resolution variations in the field-of-view, and thus presents a basic tool for image calibration of sectioned images [6].

The ultimate goal of all calibration and alignment procedures is to obtain quantitative, comparable, and reproducible data independent from the microscope system used. On the one hand, several approaches, allowing comparison of microscope systems, can be found in the literature; most of which are utilising normalized or arbitrary units [7, 8]. On the other hand, calibration procedures calculating real numbers of photons are very uncommon. Thus, fair comparisons between different systems are rare. With the sectioned image property (SIP) approach, Brakenhoff et al. reported the first descriptive calibration procedure for confocal microscopes [9]: using a spin-coated homogenous fluorescent layer as a sample, an axial image stack is acquired. The Gaussian-like axial profile of this image is then used for calibration. The authors used single basic properties of this profile like the point of the maximal value and the first and the last point where the profile crosses the half maximal value to describe the amplitude (*A*), the axial position (*z*_0_), the offset (*I*_0_), the optical sectioning capability as full width at half maximum (FWHM) of the intensity profile, and a skewness parameter, calculated by the centricity of the maximal value between the two half maximal values. This concept was successfully established [6, 10, 11], and an ImageJ plugin was provided (https://sils.fnwi.uva.nl/bcb/sipcharts/SIPchart.html). However, in experimental samples where axial profiles show noisy data, the suggested approach of using the maximal value, or the first data point matching any criteria (such as half maximal values) might deliver only assumptions on the sectioned image property (SIP) parameters. To overcome this problem, we utilized different mathematical models to fit the recorded axial profiles and gain additional information from the fit parameters [12]. As we allow to apply different fit models, we are able to describe the axial distribution within the PSF. Furthermore, the fit approach provides more robust results, in particular because the noise does not have such a high impact on the SIP parameters. Moreover, a fit approach facilitates the selection of other types of calibration samples. Thus, one is not restricted to a fluorescent layer anymore. Since the production of the spin-coated homogenous (multicolour) fluorescent layer is technically very difficult [6, 13], we suggest to utilize a sample of fluorescent solution. Such a solution is easily prepared, cheap, and can be reproducibly created with various colours at defined dye concentrations. In addition, the border between glass and solution gives a nice sample for the fit-based extended SIP (eSIP) chart. It also solves the problem of the refractive index above the spin coated layer, which is in most cases simply air, mounting media, or glass.

The concept of fluorescent solution as calibration sample requires only slight modifications of the SIP parameters: first, the axial profile does not allow a direct access for the optical sectioning capability, as the lookup of the FWHM; we utilize the steepness of the intensity increase phase of the profile as a measure for the corresponding FWHM of a thin fluorescent layer. Second, the solution profile hardly allows quantifying a skewness of the profile. Finally, the solution approach allows the estimation of the penetration depth, which is expressed by a length constant *LC* (the intensity change with penetration depth).

In the present study, we explain the concept of the eSIP approach, and we show the similarity between the solution and thin layer concept with regard to the determination of the illumination profile and the field resolution. We furthermore demonstrate that the use of a calibration solution makes it possible to account for optical aberrations. Obtaining the recorded number of photons from the photon statistics allows to control the microscope performance over time and to compare the sensitivity of different microscope setups. Finally, the eSIP approach can also be used to optimize the objective’s correction collar and collimator settings according to experimental requirements on a daily basis.

## Material and Methods

### Mathematical models

A first approximation to the axial intensity profile *I*(*z*) of a thin homogeneous fluorescent layer is a Gaussian function:

$$I\left(z\right)=I_{0}+Ae^{-4\;\text{ln}\;2{\left(\frac{z-z_{0}}{\omega_{FWHM}}\right)}^{2}}$$ (1)

**Fig 1:**
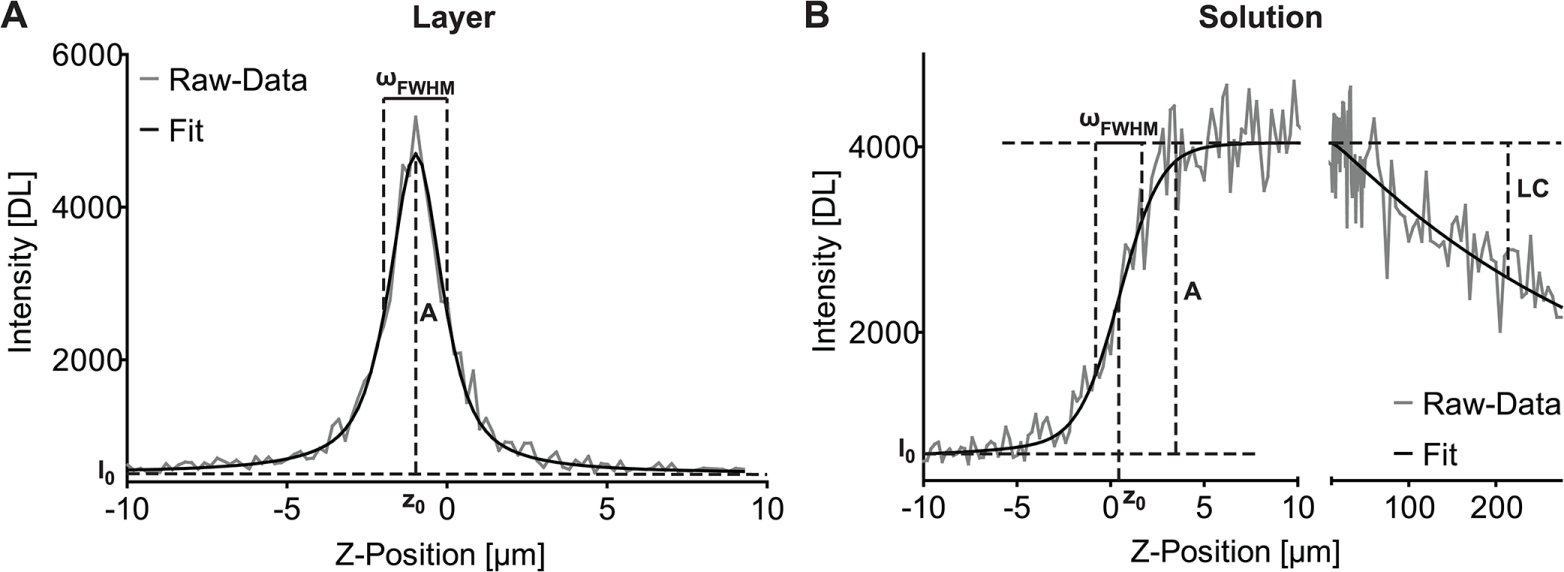
Schematic representations of the two basic calibration concepts. The fit approach to measure eSIP parameters using a homogenous fluorescent layer (**A**) reveals the parameters amplitude (*A*), full width at half maximum (*ω*_*FWHM*_), the axial position (*z*_0_) and the offset (*I*_0_). The skewness parameter and the Lorentz-Gauss fraction are not shown. If the solution-based sample is used (**B**) the steepness of the profile can also be expressed by *ω*_*FWHM*_ (compare Eqs 5–9.), and instead of the skewness parameter we included the length constant (*LC*). Example data are shown in grey, and an appropriate fit is shown in black.

*I*_0_ is the intensity offset, *A* the maximal profile intensity, *ω*_*FWHM*_ is the axial resolution reflected by FWHM, *z*_0_ the axial position of the intensity maximum (Fig 1). Accounting for the fact that the axial intensity profile will not be perfectly symmetric due to slight refractive index mismatch, we approximated the skewness of the profile by introducing a skewness factor *s*, which leads to a skewness corrected axial position *z*_*s*_ for the skewed profile:

$$z_{s}=z\cdot e^{s\cdot z}$$ (2)

A perfect Gaussian z-profile will only be obtained at homogeneous illumination of the objectives back-aperture. Most state of the art commercial laser scanning microscopes trade off some resolution against intensity: the lasers have a Gaussian beam profile, and trying to illuminate the objective’s back aperture homogeneously means rejecting approx. 80% of the excitation light [14]. As a consequence, the actual axial intensity profile can rather be described by a Lorentzian function,

$$I\left(z\right)=I_{0}+A\frac{\omega_{FWHM}^{2}}{4{\left(z_{s}-z_{0}\right)}^{2}+\omega_{FWHM}^{2}}\;,$$ (3)

whose characteristics include a smaller maximum and wider side-lobes than the Gaussian function and thus accounts for additional signal far from the optical plane.

To address the characteristics of the illumination profile, a more robust description can be obtained from a convolution of a Gaussian and a Lorentzian function, known as the Voigt function [15]. A computationally less expensive approximation of the Voigt-function can be obtained by replacing the convolution integral by a linear combination of a Lorentzian and a Gaussian function, which is known as pseudo-Voigt function [16, 17]:

$$I\left(z\right)=I_{0}+A\left[m_{L}\frac{\omega_{FWHM}^{2}}{4{\left(z_{s}-z_{0}\right)}^{2}+\omega_{FWHM}^{2}}+\left(1-m_{L}\right){\text{e}}^{-4\;\text{ln}\;2{\left(\frac{z_{s}-z_{0}}{\omega_{FWHM}}\right)}^{2}}\right]\;,$$ (4)

whose systematic error compared to the Voigt-function is less than 1%. The Voigt function readily yields the Lorentz-Gauss fraction *m*_*L*_ ∈ [0, 1] to quantify the “degree of overfill”, such that it allows to estimate the actual intensity profiles at the back aperture.

When recording an image stack of a fluorescent dye solution instead of a thin homogeneous fluorescent layer, the axial intensity profile will be the integral over the aforementioned functions (Eqs 1, 3 and 4). As the solution profile hardly allows quantifying a skewness we assume symmetric profiles (skewness factor *s* = 0). In case of the Gaussian function, we expect the line shape of an error function. Additionally, there might be an exponential drop of the intensity profile in solution due to various reasons: A slight refractive index mismatch can never be excluded and might lead to signal reduction as well as signal increase within the solution behind the cover slip-solution interface. Even in solutions with low concentrations, fluorescence pre- and post-filter effects cannot completely be excluded, so especially reabsorption of fluorescence photons can lead to exponential intensity reduction with increasing penetration depth. The effect of different fluorophore concentrations on the length constant as well as the reabsorption effect are shown in S1 Fig. Since reabsorption is typically the main effect that influences the intensity profile within the solution, we decided to model it using a mono-exponential function. While a stretched exponential function might be suited well to incorporate all possible sources of non-constant behaviour [18, 19], we preferred the robustness of a length constant. Thus, the Gaussian approximation to an axial intensity profile of a fluorescence solution is given by:

$$\begin{array}{l} I\left(z\right)=I_{0}+A\frac{\sqrt{4\;\text{ln}\;2/\pi }}{\omega_{FWHM}^{}}\int_{z_{0}}^{\infty }e^{-4\;\text{ln}\;2{\left(\frac{z'-z}{\omega_{FWHM}^{}}\right)}^{2}}e^{-LC\left(z'-z_{0}\right)}dz'\\ \;=I_{0}+\frac{1}{2}Ae^{LC\left(\frac{LC\cdot \omega_{FWHM}^{2}}{16\;\text{ln}\;2}-z-z_{0}\right)}Erfc\left[\sqrt{\frac{1}{16\;\text{ln}\;2}}\left(LC\cdot \omega_{FWHM}^{}-8\;\text{ln}\;2\frac{z-z_{0}}{\omega_{FWHM}^{}}\right)\right] \end{array}$$ (5)

where *LC* is a length constant for the mono-exponential intensity reduction, $\sqrt{4\;\text{ln}\;2/\pi }/\omega_{FWHM}$ is a scaling factor to obtain 1 for deep penetration depths z and *LC* = 0, and $Erfc\left[x\right]=1-Erf\left[x\right]=\frac{2}{\sqrt{\pi }}\int_{x}^{\infty }e^{-t^{2}}\;dt$ is the complimentary error function. The steepness of *I*(*z*) correlates with the FWHM of the gauss function in the integral. Therefore *ω*_*FWHM*_ can be used as the steepness parameter, describing the optical sectioning capability of the microscope.

To better account for additional influences we included an offset in the exponential intensity change with penetration depth. The equations then extend to:

$$\begin{array}{l} I\left(z\right)=I_{0}+A\frac{\sqrt{4\;\text{ln}\;2/\pi }}{\omega_{FWHM}}\int_{z_{0}}^{\infty }e^{-4\;\text{ln}\;2{\left(\frac{z\prime -z}{\omega_{FWHM}}\right)}^{2}}\left[\left(1-ol\right)e^{-LC\left(z\prime -z_{0}\right)}+ol\right]dz\prime \\ \;=I_{0}+\frac{1}{2}A\left(\begin{array}{l} \left(1-ol\right)\cdot e^{LC\left(\frac{LC\cdot \omega_{FWHM}^{2}}{16\;\text{ln}\;2}-z+z_{0}\right)}\cdot Erfc\left[\sqrt{\frac{1}{16\;\text{ln}\;2}}\left(LC\cdot \omega_{FWHM}-8\;\text{ln}\;2\frac{z-z_{0}}{\omega_{FWHM}}\right)\right]\\ +ol\cdot \left(1+Erf\left[\sqrt{4\;\text{ln}\;2}\frac{z-z_{0}}{\omega_{FWHM}}\right]\right) \end{array}\right) \end{array},$$ (6)

where *ol* stands for the offset of the exponential intensity reduction.

To minimize computational load, we accept a minor systematic error by considering the exponential intensity reduction as constant over the excitation point spread function and receive for Eqs 5 and 6:

$$\begin{array}{l} I\left(z\right)=I_{0}+A\frac{\sqrt{4\;\text{ln}\;2/\pi }}{\omega_{FWHM}}\left[\left(1-ol\right)e^{LC\left(z-z_{0}\right)}+ol\right]\int_{z_{0}}^{\infty }e^{-4\;\text{ln}\;2{\left(\frac{z\prime -z}{\omega_{FWHM}}\right)}^{2}}dz\prime \\ \;=I_{0}+\frac{1}{2}A\left(\left(1-ol\right)\cdot e^{LC\left(z-z_{0}\right)}+ol\right)\cdot \left(1+Erf\left[\sqrt{4\;\text{ln}\;2}\frac{z-z_{0}}{\omega_{FWHM}}\right]\right) \end{array},$$ (7)

As with layers, also in the solution approach the Lorentzian z intensity profile accounts for inhomogeneous illumination of the objective's back-aperture. Applying the approximation for the exponential intensity change with penetration depth as for Eq 7, the Lorentzian profile for solution is approximated by:

$$\begin{array}{l} I\left(z\right)=I_{0}+A\frac{2}{\pi \omega_{FWHM}}\left(\left(1-ol\right)\cdot e^{LC\left(z-z_{0}\right)}+ol\right)\;\int_{z_{0}}^{\infty }\frac{\omega_{FWHM}^{2}}{4{\left(z\prime -z\right)}^{2}+\omega_{FWHM}^{2}}dz\prime \\ \;=I_{0}+\frac{A}{\pi }\left(\left(1-ol\right)\cdot e^{LC\left(z-z_{0}\right)}+ol\right)\cdot \left(\frac{\pi }{2}+\text{arctan}\left[\frac{2\left(z-z_{0}\right)}{\omega_{FWHM}}\right]\right) \end{array}.$$ (8)

Also in the case of the Lorentzian approximation *ω*_*FWHM*_ can be used as parameter for the steepness of the intensity profile.

Analog to Eq 4 for solution the pseudo-Voigt profile can be assumed as the superposition of the Lorentzian and Gaussian profile (Eqs 7 and 8) as:

$$I\left(z\right)=I_{0}+A\left(\left(1-ol\right)\cdot e^{LC\left(z-z_{0}\right)}+ol\right)\;\left[\begin{array}{l} \frac{m_{L}}{\pi }\left(\frac{\pi }{2}+\text{arctan}\left[\frac{2\left(z-z_{0}\right)}{\omega_{FWHM}}\right]\right)\\ +\frac{\left(1-m_{L}\right)}{2}\left(1+Erf\left[\sqrt{4\;\text{ln}\;2}\frac{z-z_{0}}{\omega_{FWHM}}\right]\right) \end{array}\right]$$ (9)

### The detector amplification

Most existing photon detectors like photomultiplier tubes do not yield the number of actually detected photons, but provide intensity information *I* in digital levels (DL) being comprised of detected photons *p* and the photon conversion factor *CF* as *I* = *CF* ⋅ *p* + *I*_0_. Employing the Poisson-distribution of the detected fluorescence photons and the property of the Poisson distribution whose variance var(*p*) equals its mean 〈*p*〉, the photon conversion factor can be determined, thus allowing for the estimation of the number of detected photons.

$$\frac{\text{var}\left(I\right)}{\langle I\rangle }=\frac{CF^{2}\cdot \text{var}(p)}{CF\cdot \langle p\rangle }=CF$$ (10)

Usually, time series of a defocused image are measured, followed by a pixel-wise statistical analysis. A dark image provides *I*_0_ and the var(*I*) = *f*(〈*I*〉) is linearly fitted to obtain the photon conversion factor [20]. Since the datasets acquired with the eSIP approach provide a certain homogeneity in the optical plane, it is sufficient to calculate the variance and mean of the detected signal from laterally neighbouring pixels. We calculate the photon statistics in bins of 4 x 4 pixels. By the recording of z-stacks, we are able to map a considerable part of the detector’s dynamic range. This will only work if some images of the z-stack are recorded from well inside the cover slip.

The photon conversion factor can be used to calculate the number of detected photons per pixel. Since the photon conversion factor is depending on the detector efficiency, which in turn is wavelength dependent, a fixed microscope detector gain setting will yield different photon conversion factors for different emission channels. Therefore, this calibration has to be done for each detection channel.

### Acquisition settings

**Fig 2:**
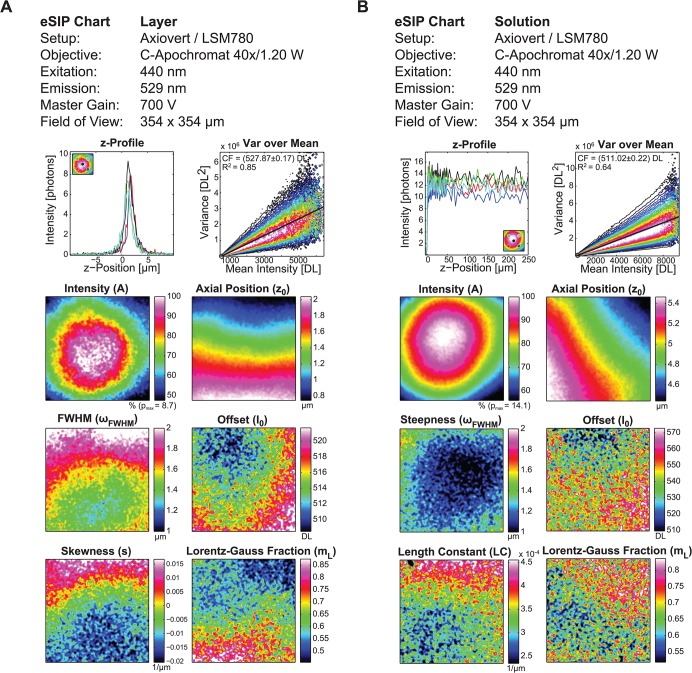
Extended section imaging property charts from layer and solution-based calibration samples. Comparable eSIP analysis using the same microscopic system with a homogenous fluorescent layer (**A**) and the solution-based calibration sample (**B**). Axial profiles (z profile) are shown in 5 different spatial positions (shown as a scheme within the plot). The 'Var over Mean' plot is scaled to maximum frequency in each Intensity bin to account for the different intensity distributions for layer and solution. The conversion factor (*CF*) is obtained by a linear fit (black line), which is 527.87±0.17 for the layer and 511.02±0.22 for the solution-based approach. The regression coefficient *R* ^2^ is given to describe the goodness of fit. All eSIP parameters are shown as 3 dimensional plots: Intensity (*A*) in percent of the maximal photon number (*p*_max_), axial position (*z*_0_) in μm, FWHM or steepness (*ω*_*FWHM*_) in μm, offset (*I*_0_) in digital levels (DL), skewness (*s*) or length constant (*LC*) in 1/μm and the Lorentz-Gauss fraction (*m*_*L*_). Additional scanning parameters: excitation 440 nm with 2% laser power, emission Channel 529 nm centre wavelength, pinhole 0.5 AU, detector gain 700V, 354 x 354 μm field of view with 512 x 512 pixels, pixel dwell time 1.58 μs, and axial spacing is in (A) 0.2 μm and in (B) sequentially 0.1 μm (10 μm around the glass/solution border), 1 μm (for adjacent 45 μm) and 5 μm (for adjacent 200 μm).To give an impression on the computational load: The analysis of the data from (B) took about 3 minutes using a scripting language (MATLAB) on a good equipped office calculator (Intel Core i7 3.2GHz 64 bit system with 32 GB). An analysis of the data from (A) using the SIP approach published by Brakenhoff et al. can be found in S2 Fig.

**Fig 3:**
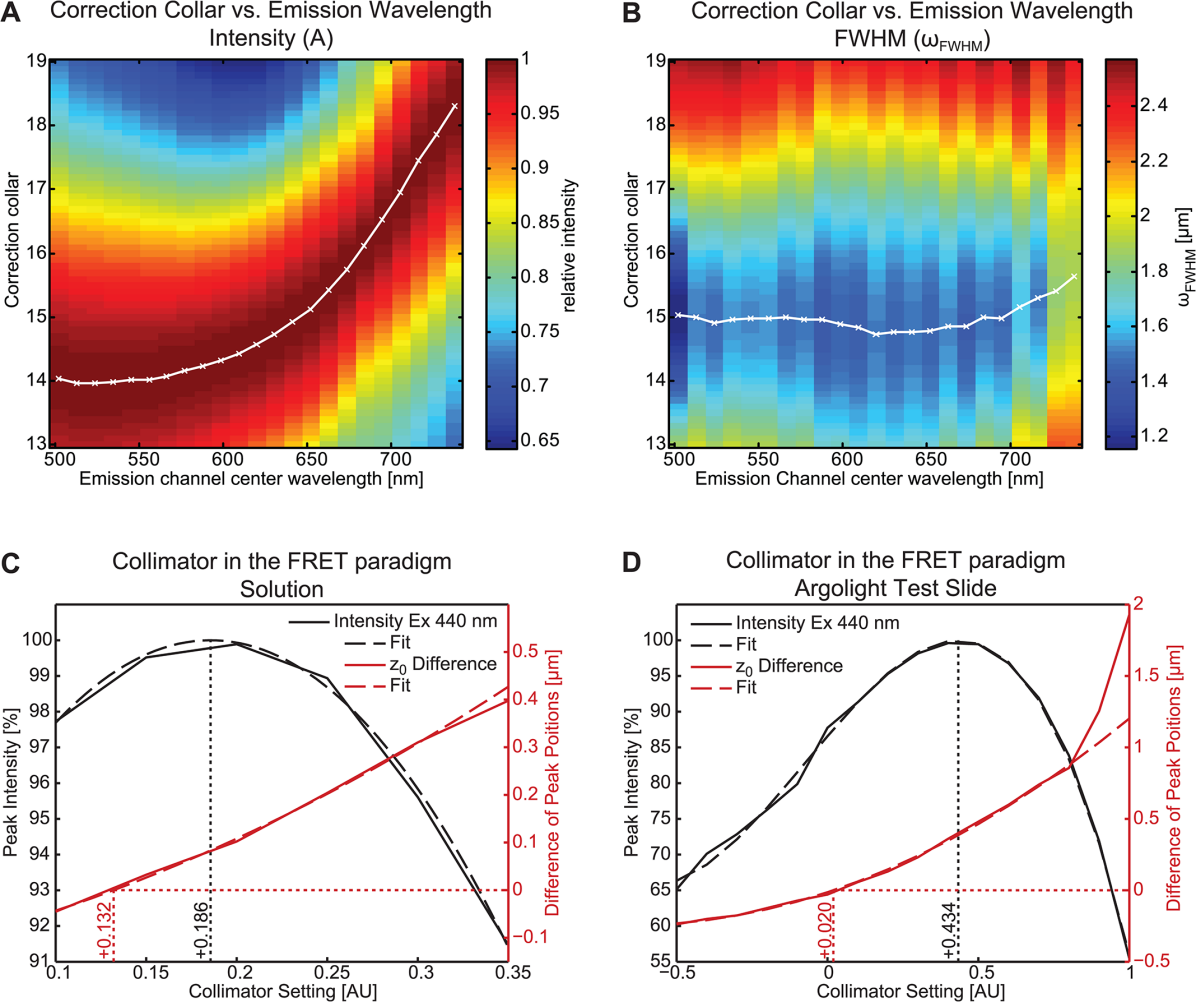
Using section imaging property parameters to optimize microscope system settings. To estimate the correction collar setting at a Zeiss LSM 510 confocal microscope, a series of eSIP measurements can be used to define the influence on single eSIP parameters. (**A**) 2d plot of the intensity parameter for correction collar settings of a 40x/1.2 W C-Apochromat (Zeiss) ranging from 0.14 to 0.19 obtained at a wavelength range from 500 nm to 740 nm with pinhole setting 1.0 AU is shown. The data is derived from a 115 x 115 μm centre region of each plane. Although using an Apochromat the emission wavelength dependency is evident in this measurement. However the optimal settings can easily be found. In analogue fashion the influence of the correction collar was analysed based on the *ω*_*FWHM*_ parameter (**B**). Interestingly, there is no wavelength dependency for this parameter. Analysing the collimator setting in a lux-FRET paradigm with two different excitations at a Zeiss LSM 780 utilizing a 40x/1.2 W C-Apochromat objective revealed different optimal settings in respect to the observed parameter (**C and D**). In contrast to an optimization according to the maximal intensity (black), we found a different optimal setting, when the axial position difference between first (440 nm) and the second excitation (488 nm) is measured (red). For the collimator setting series we tested the solution-based approach (**C**) as well as the Argolight calibration slide (**D**).

Unless otherwise noted, experiments were carried out on an inverted and motorized microscope (Axio Observer Z.1) equipped with a 40x/1.20 W C−Apochromat objective. The attached laser scanning unit (LSM 780, Zeiss, Jena, Germany) enabled confocal imaging. For excitation, 440 nm and 488 nm lasers were used. Unless otherwise noted pinhole was set to 0.5 AU. Detailed scanning parameters are listed in the corresponding figures / figure captions. Images were recorded at 16-bit pixel depth. For analysis, they were processed in MATLAB scripts utilizing the eSIP formulas described above. As start parameters for the fitting we used the parameters obtained from the lookup approach [11]. The fluorescein/perylenediimid-layer reference layer, thickness ≈ 110 nm, *n*_D_ ≈ 1.59 was a generous gift by J. M. Zwier. The fluorescent solution used in Fig 2 is a dilution of fluorescein in distilled water (Uranine AP, *λ*_em_ = 516 nm, OD = 1.37 for 1 cm at 487 nm). To account for a wider emission wavelength range needed for Fig 3 Rhodamine 6G (*λ*_em_ = 555 nm) was added and adjusted to an equal fluorescence intensity (both fluorophores from Applichem, Darmstadt, Germany). The solution was imaged in an ibidi μ-Dish with 170±5 μm glass bottom (ibidi GmbH, Martinsried, Germany).

### Analysis of the Argolight grid structure

In general, the grid structure was imaged using the setup described in the Acquisition settings section. Z-stacks were imaged using two excitations (440 and 488 nm) and 32 emission channels spanning 411–682 nm. The emission channels with the maximal intensity (538 and 573 nm) were used for further analysis using MATLAB. Crossing points of the grid structure were found by creating a binary image morphological filtering. To quantify the distortion, these crossing points are grouped and fitted using a polynomial fit of the second order. The second order term is used to describe the distortion (0 = linear). The difference in distortion is found by comparing the crossing points of the two excitation channels.

## Results

### From layer to solution

The main advantages of a thin homogeneous fluorescent layer for the determination of microscope performance include its long term stability, optical stability, and its intuitive axial intensity profile which is easy to describe mathematically. Contrary to that, fluorescent dye solutions need to be freshly prepared and need to be protected from evaporation. Dye molecules tend to precipitate at the cover slip surface, and can neither be used for dipping lenses nor for “pseudo-sectioning” microscopes like slit-scanning and spinning disk confocal microscopy. Finally, the computational cost to numerically fit the error function is significantly higher. However, a fluorescent dye solution offers essential advantages over a thin homogeneous fluorescent layer: it can be easily prepared in reproducible manner and can contain a well-concerted set of dyes to cover the whole spectrum of interest, so that the system calibration can be performed for multiple excitation and emission channels simultaneously, depending on the experimental interest. Moreover, synthetic dyes used for fluorescence labelling can be further used for the preparation of the calibration solution, which in combination with known concentrations enables a more precise quantitative comparison. In addition, the intensity profile in the solution contains dye-specific information about the correct adjustment of the correction collar and refractive index mismatch.

Comparing the eSIP approach for a confocal microscopy system using a spin coated fluorescent layer and a solution-based fluorescein sample eSIP parameters show values in comparable ranges and distributions (Fig 2). In the depicted comparison only one emission channel (centre wavelength 527 nm) is used. Charts were imaged using the same detector gain of 700 V, which in turn leads to similar conversion factors shown in the 'Var over Mean' plot calculated as the slope of the linear fit (527 and 511 DL/photon) [20]. However, the regression coefficients *R* ^2^ of the linear fits do differ. The reduced *R* ^2^ of 0.64 for the solution-based sample might be due to the higher amount of intense pixels within the solution. In the z-Profile plot the axial intensity distributions are shown for 5 exemplary bins (depicted in the schematic legend). The noise charged profiles seen in the z profile plot of Fig 2 still lead to a good data outcome. This clearly shows one of the advantages of the eSIP approach, were parameters are received from a fit. The intensity value *A* itself cannot be compared since different sample types are used. However, the distribution of the amplitude visualizes insights in the optical calibration. *z*_0_ clearly depends on the sample flatness itself and the sample holder adjustment; the direct comparison can indicate holder specific properties. The values for *z*_0_ do differ in the origin but show the same range. Since FWHM for layer and the steepness parameter for solution are analogous, both predict the axial resolution *ω*_*FWHM*_. In the comparison of layer and solution the axial resolution is worse for the layer, which in fact is expected for the use of a water immersion objective for the mounted layer sample. The offset *I*_0_ shows background information in the field of view, resembling either optical perturbations or calibration sample inhomogeneities–a direct comparison of the two samples is not possible. The skewness of the layer profile *s* as well as the length constant *LC* of the solution can indicate refractive index mismatches. In case of solution, dye concentration dependent reabsorption can also influence the length constant *LC* (see S1 Fig). The last parameter, the Lorentz-Gauss fraction *m*_*L*_, which describes the beam profile homogeneity of the back aperture with a value 0 for a perfectly homogenous beam in the back aperture (axial Gauss profile in the sample plane) and value 1 for a gauss shaped beam profile in the back aperture, does not show a comparable distribution. Nonetheless, the Lorentz-Gauss fraction values are in a comparable range of around 0.65 for the chosen microscope setup. Some of the parameters (especially *ω*_*FWHM*_, *I*_0_ and *m*_*L*_) do appear noisy. This is due to the elected low fluorophore concentrations. By acquiring higher number of photons much better signal to noise ratios can be obtained (compare S1 Table and S3 Fig). This can be achieved by increasing laser power, acquisition time or fluorophore concentration. The latter however will especially for the solution-based sample lead to reabsorption effects and will reduce the penetration depth, which in turn leads to a decreased length constant *LC* (see S1 Fig). Taken together, the solution-based sample indeed represents a suitable alternative to the spin-coated layer. Although some differences in outcome still exist, the eSIP calibration using solution can be easily conducted in reproducible manner.

### Application of the eSIP approach

The eSIP approach can be employed to characterize complex microscopic setups and their optical properties and capabilities for quantification approaches. The information obtained can then be used for setup adjustment and calibration. The most obvious adjustment options which benefit from the eSIP approach include correction collar, collimator, check for pinhole alignment and optimisation of the sample holder. For the latter the axial position parameter can be directly utilized for mechanical correction of the sample holder. Correction collar and collimator can be adjusted after analysis of an eSIP measurement series at various correction collar and collimator settings. To illustrate the capabilities and relevance of the eSIP approach, Fig 3 shows representative measurement sets to adjust correction collar (Fig 3A and 3B) and collimator (Fig 3C). In these adjustments the utilized eSIP parameter can vary dependent on the system, the dedicated experiments or the notion of the operator. The most reasonable parameters for this task are intensity *A* and the axial resolution *ω*_*FWHM*_, but also the axial position *z*_0_ is of interest here. In particular, if different channels are used for ratiometric approaches, perfect overlap of the axial profiles is crucial. In case of correction collar adjustment using the solution-based approach surprisingly we found a clear emission wavelength dependency at the maximal intensity using a C-Apochromat 63x/1.2 W objective from Zeiss (Fig 3A). Observing *ω*_*FWHM*_, we did not find any wavelength dependency (Fig 3B). Thus optimal settings for intensity and axial resolution do not coincide and the optimal setting clearly depends on the experimental need. The wavelength dependency for the maximal intensity impressively shows the importance of matching refractive indices for confocal microscopy. The eSIP approach allows finding the optimal setting, according to the experimental requirements, *e*.*g*., the emission channel chosen. The adjustment of the collimator setting (which is typically required only for 'invisible' laser lines) was performed for the alternating excitation with two laser lines [21], which is the basic concept of quantitative linear unmixing Förster resonance energy transfer (lux-FRET). In case of 440 nm / 514 nm excitation, which is a standard regime for Cyan and Yellow fluorescent protein like FRET pairs, the 440 nm collimator has to be adjusted to match *z*_0_ of the 514 nm excitation [22]. In contrast to the standard adjustment approach according to maximal intensity, we additionally analysed the axial positions of the emission channels at 440 and 514 nm excitation and plotted the difference of *z*_0_ (Fig 3C and 3D) for a representative emission channel, as no differences in *z*_0_ was found for different emission channels. In the case of the solution-based approach, the two eSIP parameters did show a clear difference in optimal collimator setting: 0.132 AU and 0.186 AU for *A* and *z*_0_ difference, respectively. In summary, these results demonstrate that the eSIP approach can easily be used to optimize microscope settings. However, experimental needs may lead to different optimal values.

### Comparing setups

We could show that the eSIP approach can be used to characterize optical sectioning properties of a microscope system and can also be utilized to adjust system settings. In addition, the use of the eSIP approach can facilitate the comparison of experimental results from different microscope setups. Our goal was to provide a tool to allow the comparison of quantitative measurements from different setups and thus to allow for standardization in the field of quantitative microscopy. To elucidate the potentials of the eSIP approach we prepared a series of measurements with different objectives at the same microscope.

The results of the eSIP application were obtained at a Zeiss LSM 780 and are summarized for various objectives in Table 1. According to the objectives’ magnification and numerical aperture, in these measurements different field of views were observed, and the number of collected photons per pixel varied according to the size of the confocal volume. For example, the 10x/0.30 EC Plan Neofluar objective is able to collect a significant higher number of photons compared to the 40x/1.2 W C-Apochromat objective since its confocal volume is much larger. The axial resolution *ω*_*FWHM*_ of the optical system is in a comparable range with theoretical resolution taking into account the NA and pinhole settings. In case of the length constant (*LC*) application of water as an immersion media (40x) does not impair the *LC* value within the water-based fluorescent solution. In contrary, acquiring the z-stack of the aqueous fluorescent solution with the 63x oil immersion objective gives a significant drop of intensity of about 61% at 100 μm penetration depth, which is caused by the substantial refractive index mismatch and represented by a high *LC* parameter.

**Table 1:** Comparison of eSIP parameters using different objectives at a Zeiss LSM 780.

Objective	A^a^ [photons]	ω_FWHM_ [μm]	LC [mm^-1^]
10x/0.30 EC Plan−Neofluar	6.86 ± 1.0	15.24 ± 1.7	0.34 ± 0.08
20x/0.8 Air Plan-Apochromat	6.39 ± 0.43	3.10 ± 0.49	2.64 ± 0.27
40x/1.20 W C−Apochromat	1.11 ± 0.11	1.61 ± 0.22	0.12 ± 0.08
63x/1.40 Oil DIC Plan−Apochromat	0.32 ± 0.06	0.75 ± 0.24	9.52 ± 0.47

Measurements are conducted using the solution-based calibration sample and an excitation at 440 nm with 5% laser power and pinhole setting 0.5 AU.

^a^ A: Amplitude or maximal intensity. The differences in intensity also originate from the big differences in the field of view. Same settings for the zoom result in following field of view sizes. 10x: 1417×1417 μm, 20x: 708×708 μm, 40x: 354×354 μm, 63x: 225×225 μm. All data were measured using 512x512 pixels resulting in different pixel sizes.

Taken together our novel eSIP approach could be used to obtain detailed characteristics of the given setup which then in turn can be used to compare quantitative results.

### A possible alternative to layer and solution

**Fig 4:**
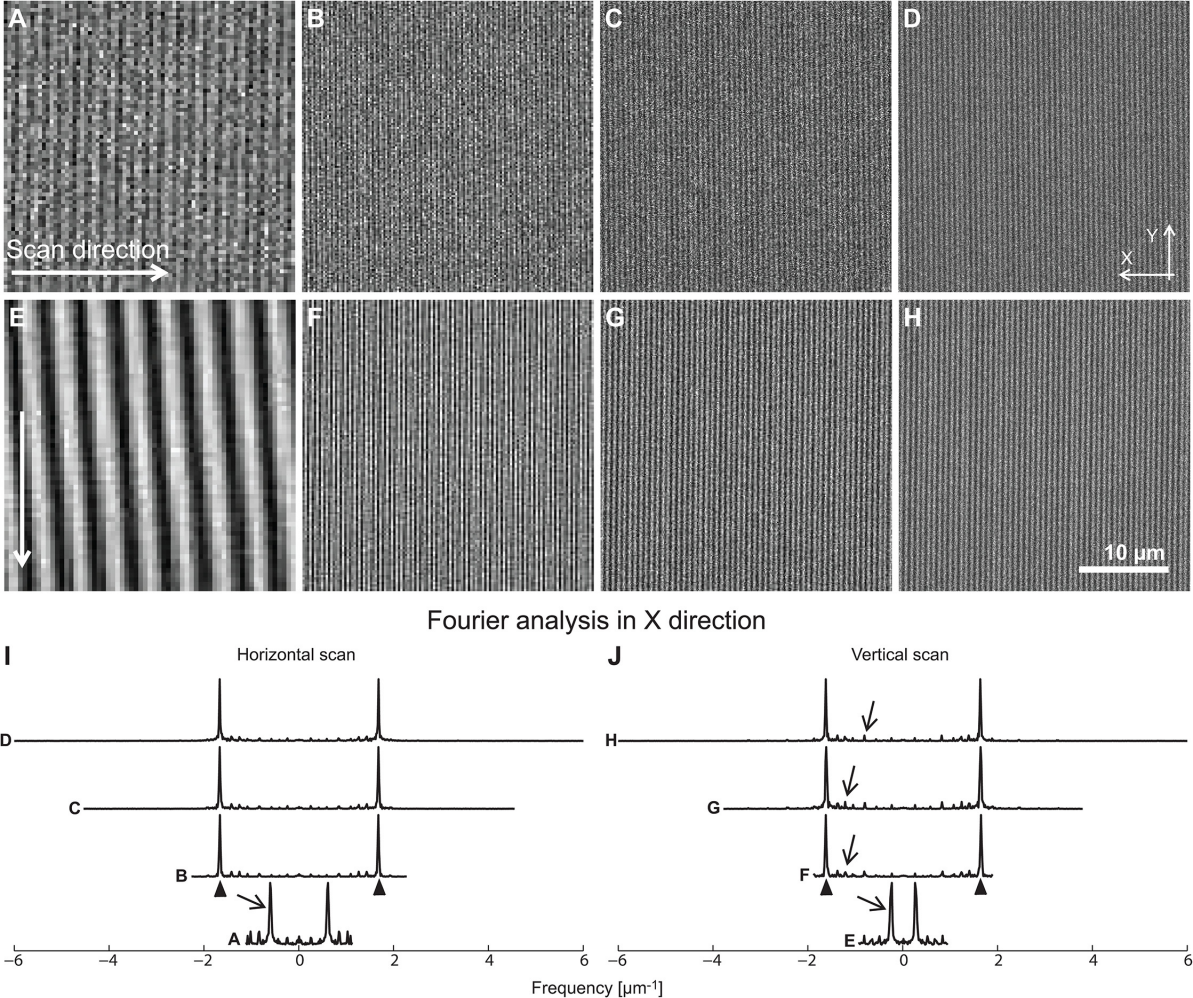
Moiré artefacts using structured calibration samples. Example images of maximum projections, taken from a structured non-homogenous layer (Argolight calibration sample). The depicted images show the same field of view of a vertical grid structure (the “homogenous pattern” of the Argolight slide), which were either scanned perpendicular to the grid (**A-D**) or in a parallel fashion (**E-H**) with a decreasing pixel size (increasing number of pixels). A Fourier analysis in x direction of the images (**I and J**) reveals several instances of moiré artefacts (arrow) in addition to the frequency of the grid itself (arrow head). At low resolution (**A and E**), these artefacts are dominating the images; the grid structure itself becomes invisible.

Although the preparation of a fluorescent solution-based standard reduces technical effort compared to the production of a thin fluorescent layer, the effort to create a defined standard for calibration could still be improved by a highly standardized alternative that is commercially available. Several aspects should be taken into consideration. The calibration sample has to be highly stable over time in order to allow for quantitative comparison of different experiments. Moreover, the manufacturing of calibration samples must be highly reproducible over various product charges to allow for the comparison between different laboratories. The so far only commercially available product which addresses to fulfil the requirements mentioned above (*e*.*g*., the production is standardized and the long term stability of fluorescence is guaranteed for five years) is the Argolight calibration slide. We therefore evaluated an ARGO-M slide (standard version of mid 2014) [23] for its calibration properties and its usability for the eSIP layer approach. These slides contain different line-style patterns imprinted into the slide (http://argolight.com/argo-m-standard-slide/). The basic element of all the structures within the calibration sample is a tube section whose diameter is about 600 nm, length about 4 μm (FWHM) and wall thickness about 250 nm (S4 Fig). This resembles a very complex axial profile. Scanning through the object is not analogous to a homogeneous fluorescent layer sample; it is better approximated by the convolution of a rather large structure, which increases intensity with increasing tube length. Since the structures provided are available at a defined length between 4 and 10 μm, the z-profile of the structure used requires additional fit models. All calibration structures are built up by these tubes and its line shape representative, a double line. Thus, even the tightest structures aligned like a fluorescent square were not recognized as homogeneous under standard confocal conditions. While testing this non-homogenously striped structure as a potential alternative for a thin fluorescent layer, we observed Moiré artefacts, derived from lateral frequency differences between the structure width and the 'pixel width' of the point scanning confocal microscope, similar to a beating (Fig 4). More specifically, the Moiré effect is more pronounced when the pixel size is similar to the line period, i.e. about 600 nm, for a scanning parallel to the lines.

**Fig 5:**
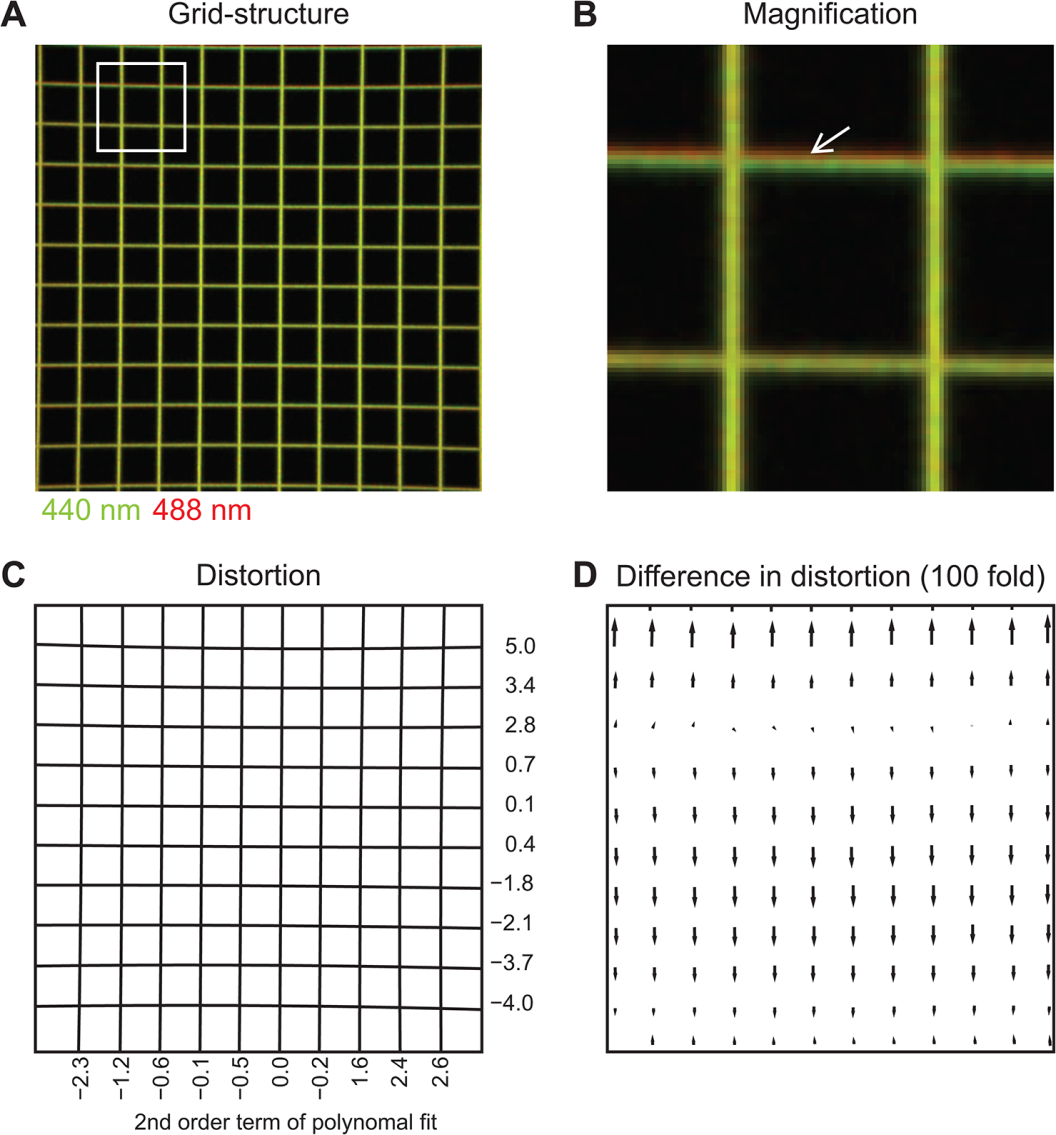
Gaining information on X and Y from a commercially available calibration sample. Neither the layer- nor the solution-based eSIP approach provide information about the lateral imaging properties. Utilizing defined fluorescent structures makes it possible to describe aberrations in these dimensions, too. The grid structure on the commercially available Argolight slide was imaged using two excitation wavelength (440 nm and 488 nm) and maximized field of view (**A**). The analysis of this grid structure revealed strong distortions which can be quantified using second order polynomial fits. To depict this aberration, the second order term (multiplied with 10^5^) is shown next to the corresponding fit (white lines) for the first excitation (**B)**. The difference in the two excitations is depicted in **C** as vectors at the position of grid crossings reflecting the direction and the size of the shift (the size of the arrows are multiplied by 100 for visibility).

Although the ARGO-M slide we tested is not an optimal sample for the eSIP approach as it is, the ARGO-M slide with its defined structures can be utilized easily to include an evaluation of lateral aberrations (Fig 5): the distortion can be quantified by a polynomial fit (second order) to estimate the linearity of the grid structure. A clear distortion towards the outer areas of the field of view is evident (Fig 5B). Comparing the grid structure upon excitation at different wavelengths clearly demonstrates that the aberrations obtained in this optical system are wavelength-dependent (Fig 5C).

Further, the current ARGO-M substrate is specified with a refractive index of 1.60 at 589 nm. In order to correct for the refractive index mismatch, the structures are placed at a 'compensating' z-level (personal communication). Still, the ARGO-M is a perfect tool to simply visualize lateral aberrations, which is not possible with the SIP calibration samples thin fluorescent layer and fluorescent solution.

## Discussion

### The extended SIP approach

Brakenhoff *et al*. [9] introduced a powerful tool for the calibration of optical sectioning microscope setups using thin fluorescent layers. This approach is based on the characterization of the axial profile observed, leading to an intuitive analysis with low computational effort. It was developed further [10, 11] and was applied successfully in a number of publications [24–27]. In order to make this approach available for the researcher, an ImageJ plugin has been made available at https://sils.fnwi.uva.nl/bcb/sipcharts/SIPchart.html. With the eSIP approach we now significantly extend this idea by (I) introducing a fit approach and (II) using fluorescent solution as calibration sample, thus we provide a more general application. In cases where variations due to noise and effects caused by inhomogeneous back aperture illumination cannot be neglected, applying the fitting approach allows to obtain eSIP parameters with at least similar accuracy compared to the SIPchart approach. Analysing toy data which show comparable variations in fit parameters as shown in Fig 2 illustrates the accuracy dependence with photon number for both approaches whereas the fit approach lead to better results for the used input parameters (S1 Table and S3 Fig). Using the eSIP approach, fitting different distribution profiles is possible like the profiles generated from spin coated fluorescent layers and fluorescent solutions. In addition, the back aperture is characterized for illumination homogeneity, eSIP charts provide multiple parameters characterizing the optical and technical properties of the microscope setup. In addition to the evaluation of the axial intensity profiles for each pixel bin, its variance is used as described in the methods part to obtain the conversion factor from photons to digital levels. In Fig 2 we presented two exemplary eSIP charts demonstrating that the solution approach provides a similar quality as the layer approach. With the help of the intensity distribution map, illumination inhomogeneities are visualised. The alignment of the optical system is reflected by a centred and symmetric intensity profile, which can be used as a field-normalisation in data analysis [10]. Complementing the intensity map, the axial resolution map serves best to describe optimal microscope settings and allows for correction collar and collimator adjustment (Fig 3). The axial position describes the orientation of the sample in the field of view. Big tilts of the sample plane will also lead to artefacts like distortions with the lateral resolution. In the present study we obtained differences in axial position of about 1 μm in a field of view. Assuming a perfectly flat cover slip surface this reflects a tilt of 300 μm in a 10 cm sample holder, a value which is not easy to be optimised in most available sample holders. In addition to the sample holder correction, the axial position parameter can be also used as an alternative to the intensity and the axial resolution to optimize settings for best confocal overlap when measuring with different excitation wavelength (Fig 3C and 3D). This is obligatory for pixel-based, quantitative imaging approaches, like in experiments using FRET-based biosensors [22], ratiometric indicators, colocalization analysis, and other ratiometric approaches. The skewness parameter (available for the thin fluorescent layer) will show refractive index mismatches [28] or a correction collar that is not optimally adjusted [29], both would lead to wrong depth interpretations and dramatically reduced penetration depth [28, 30]. In the solution-based approach where we did not include skewness, the refractive index mismatches can be noticed even easier by the LC. If the correction collar is perfectly adjusted and the refractive indexes do match, a value of zero for the LC is expected [31] and can also be found in the data presented. The LC parameter however is also affected by high fluorophore concentrations (due to reabsorption and scattering in the media). The Lorentz-Gauss fraction, which is novel for this eSIP approach describes the illumination of the back aperture. A Gaussian distribution (*m*_*L*_ = 0) in the axial profile of the sample stands for a homogenously illuminated back-aperture whereas a Lorentzian distribution (*m*_*L*_ = 1) will be created by illumination with a Gaussian beam profile [32]. The data presented clearly show that a homogenously illuminated back aperture and thus a Gaussian axial profile of the calibration samples cannot be found (Fig 2). Assuming that the microscope manufacturers try to find the best compromise for various configurations, a homogeneous back aperture profile can only be realized by massive over-illumination of the back-aperture. In such cases, only a fraction of the excitation laser energy would be transferred through the objective. For example, Zeiss allows for back-aperture filling only in FCS mode, called pupil filling factor. Leica allows for a 6 fold over-illumination.

### The solution-based eSIP approach

The transition from a thin fluorescent layer-based calibration sample towards a solution-based approach has a couple of advantages as well as some minor disadvantages. A solution-based sample is easy to created, and it is easy to optimize for the experimental need (fluorophore, multicolour sample, dilution series, etc.). However, some fluorescent dyes tend to precipitate in aqueous solutions at the cover slip, which should be avoided. It is of course possible to include this precipitation into the fit models, but it would increase the computational effort and would introduce additional fit parameters which reduces the accuracy of other parameters, because the degrees of freedom are increased. Further, evaporation of the sample solution has to be avoided, as this will change fluorophore concentration over time. A major concern is that the solution-based approach will not work for ‘semi’-confocal optical sectioning microscopes with certain cross-talk (*e*.*g*., Spinning Disc) or for concepts like structured illumination [33]. Nevertheless, until now adequate thin fluorescent layers are not available commercially and are quite difficult to manufacture as they require experience in spin-coating [6, 13]. The solution-based sample gives the opportunity to always create a fresh calibration sample using optical density measurements to overcome bleaching or evaporation effects. This enables not only the comparison over time but also at different places using different calibration samples with comparable characteristics. In the solution-based approach, the eSIP skewness parameter *s* was not introduced; refractive index mismatches, however, can be perfectly noticed by the *LC* parameter, which can be interpreted as the penetration depth of the imaging system.

In our study, we were also able to evaluate a commercial calibration sample provided by Argolight in the context of the eSIP approach [23]. Although it did not fully match the requirements for our eSIP approach, we think it could be a good approach for the assessment and calibration of lateral imaging features in microscope systems. We suggest a state-of-the-art general standard of microscope calibration routine should be a combination of the eSIP approach with a calibration routine that provides information on the X and Y resolution and aberration. This could be a bead-based routine [3] or a novel approach using defined fluorescent structures to include field-dependent information (compare Fig 4) like the Argolight slide [23]. An ideal calibration sample for a combined approach would be a glass surface with a defined grid engraving overlaid by fluorescent solution. By this, one could easily combine our eSIP approach with the possibility of obtaining field-dependent lateral information. Only in combination and on a regular base it will be possible to develop a complete comprehension of the microscopic system and to optimise the system for the experimental need, which is a requirement for pixel-based, quantitative microscopy.

## Supporting Information

S1 Fig Effects of fluorophore concentration on the length constant LC. Axial profiles from eSIP measurements of fluorescein solution with different concentrations (**A-C**). The concentrations are derived from a diluted stock solution (1:1000, 1:100 and 1:10) and fluorophore density is measured using a fluorescence spectrometer (OD). As fluorophore density increases, the LC parameter increases accordingly. An analysis of the fluorescence emission spectrum for different z-positions of the 1:10 solution shows a clear reabsorption effect (**D**). The deeper the penetration, the stronger short wavelength parts of the emission spectra are reabsorbed. This leads to a spectral shift as emission light is postfiltered by excitation of the fluorophore. The depicted spectra are normalized for the wavelength range 560–580 nm.
(EPS)

S2 Fig SIPchart of the layer data using the SIPchart ImageJ plugin. SIPchart generated with the ImageJ plugin from Norbert Vischer based on the SIPchart approach published by Brakenhoff et al. The data used for this analysis is identical with the layer data presented in Fig 2A. In general, the plugin produces comparable results but a reduced parameter set. The plugin utilizes a bin of 8x8 pixels reducing noise and spatial resolution. The intensity values are most likely derived from a conversion from 16 to 8 bit and reflect digital levels.
(PNG)

S3 Fig Toy data–Accuracy and Precision, obtained by the SIPchart lookup approach and the eSIP fit approach. Figures represent the accuracy (black) as deviation of the mean from input parameter and the precision (red) as standard deviation (STD) of parameters obtained from toy data (S1 Table). The scaling is adjusted for each plot to allow direct comparison of accuracy and precision. (**A**) Intensity errors are provided as relative error. The SIPchart lookup approach provides too high mean intensities at low photon numbers but slightly lower at high photon numbers. At the later condition, however, the accuracy is within one STD. The fit approach for layer and solution provides increasing accuracy with increasing intensity (photon number). The decrease of the STD with photon number follows power law in all cases. In case of the layer the used input parameters both approaches give comparable STD of maximum intensity. Using the solution sample seems to be more precise in this case, which might be due to the number of intense data points. (**B**) The accuracy of FWHM gets improved with increasing intensity. The SIPchart lookup approach converges to a slightly too high FWHM for the used input parameters. The deviation, however, is still within one STD. Similar to the relative STD of the intensity the precision of the fitted FWHM follows power law. (**C**) The axial position can be obtained with very high accuracy for all methods compared to the used z-spacing, which was 0.2 μm for the layer and 0.1 μm for solution. Due to the concept of the layer, where two edges are used for z_0_ determination, especially at low photon numbers the accuracy is notoriously better than the solution approach with only one edge for z_0_ determination. For the lookup concept, the precision of z_0_ highly depends on the z-spacing. Therefore, contrary to the eSIP fit approach, also at very high intensity levels the STD of z_0_ does not further improve. (**D**) The STD of the offset (I_0_) for the SIPchart lookup approach mainly depends on the number of values not influenced by the layer. This can be optimized to receive comparable STD compared to the eSIP fit approach, if required. (**E**) To compare the skewness parameter of both layer approaches with different conditions we calculated the lookup skew values from noise free Gauss profiles calculated by Eqs 1 and 2. The conversion factor is of about -2 (skew = -2*s) for a wide range of skew values and is used to convert the SIPchart skew values in **F**. (**F**) The obtained variation in the skewness parameter reveal the relative poor accuracy of the skewness parameter, also in the eSIP fit approach. For a relative STD of 100% an intensity of more than 20 photons (peak intensity) are required. Due to the z spacing the SIPchart lookup approach never reaches 100% STD for the chosen input parameters.
(EPS)

S4 Fig Representation of the basic structuring element of the Argolight slide. Three dimensional illustration of the basic structuring element from the Argolight slide. In this illustration, the cigar-like tubular shape is visible. The image stack was acquired from the stair-like structure on the calibration slide using a Zeiss LSM 780 confocal microscope and the Plan-Apochromat 63x/1.40 Oil objective at an excitation wavelength of 440 nm. The image stack was evaluated, and the illustration was created using Bitplane Imaris software.
(EPS)

S1 Table Toy data—Mean and standard deviation, obtained by the SIPchart lookup approach and the eSIP fit approach. To estimate the error of the fit parameters and to compare the eSIP fit approach with the SIPchart lookup approach we generated toy data by calculating gauss profiles (Eq 1) for layer and intensity profiles according to Eq 5 for solution. Maximal photon counts varying from 1 to 1000 photons and applying the corresponding Poisson noise and an additional Gaussian noise corresponding to the readout noise of the Zeiss LSM Quasar detector at detector gain 700 was applied. The following parameters have been used: data points layer (*z*), from -10 to +10 μm with 0.2 μm spacing; data points solution (*z*), from -5 to +5 μm with 0.1 μm spacing, from 6 to 50 μm with 1 μm spacing, from 55 to 200 μm with 5 μm spacing (same as in solution experiments); FWHM (*ω*_*FWHM*_), 1 μm; axial position (*z*_0_), 0 μm; offset (*I*_0_), 500 DL; photon conversion factor, 500 DL/photon, layer: skewness (*s*), -0.025, solution: length constant (*LC*), 0. We introduced an additional variation in the axial position (*z*_0_) to avoid permanent coincidence of Gauss maximum and half-maximal values with pixel bins. In correlation to the settings in the presented work a 4x4 bin was used for analysis. Mean values and standard deviation (STD) were obtained from 500 simulations per intensity level. For the intensity (*A*) relative errors are provided as STD/mean. The error dependence with photon number is illustrated in S3 Fig. **SIPchart lookup approach, layer:** The Intensity (*A*) was found by searching for the brightest value. Since the z spacing is rather small compared to FWHM, A is notoriously too high at low photon counts. Contrary, for high photon counts it is too low. FWHM was determined by a line approximation between most outlaying data points above and the neighbouring data points below half maximal value. The axial position was found as the position of maximal intensity, so the accuracy directly correlates with z spacing. The offset value was estimated as average from ten values most distant from z_0_. The skewness was obtained (according to [11]) as skew = (b-a)/(a+b) (compare S3 Fig F), which deviates from the eSIP approach definition Eq 2 by a factor of about -2. **eSIP fit approach, layer:** Parameters were obtained by fitting Eq 1 to the toy data. The look up parameter were used as start parameters for the fit. As expected, an improvement of the accuracy and lower standard deviations of the fit parameters were obtained. **eSIP fit approach, solution:** Parameters were obtained by fitting Eq 5 to the toy data. The length constant *LC* was not fitted. The accuracy as deviation of the mean from the input parameter and the precision in terms of STD are illustrated in S3 Fig. Unless toy data are only provided for one most relevant parameter set, this demonstrates the quality of all approaches, whereas the eSIP fit approach for solution reaches at least results of the same quality as the SIPchart approach for layer.
(PDF)
