# Supplementary figures and images for: eSIP: A Novel Solution-Based Sectioned Image Property Approach for Microscope Calibration
Source: PLoS One. 2015 Aug 5;10(8):e0134980. doi: 10.1371/journal.pone.0134980 (PMC4526552; doi:10.1371/journal.pone.0134980)

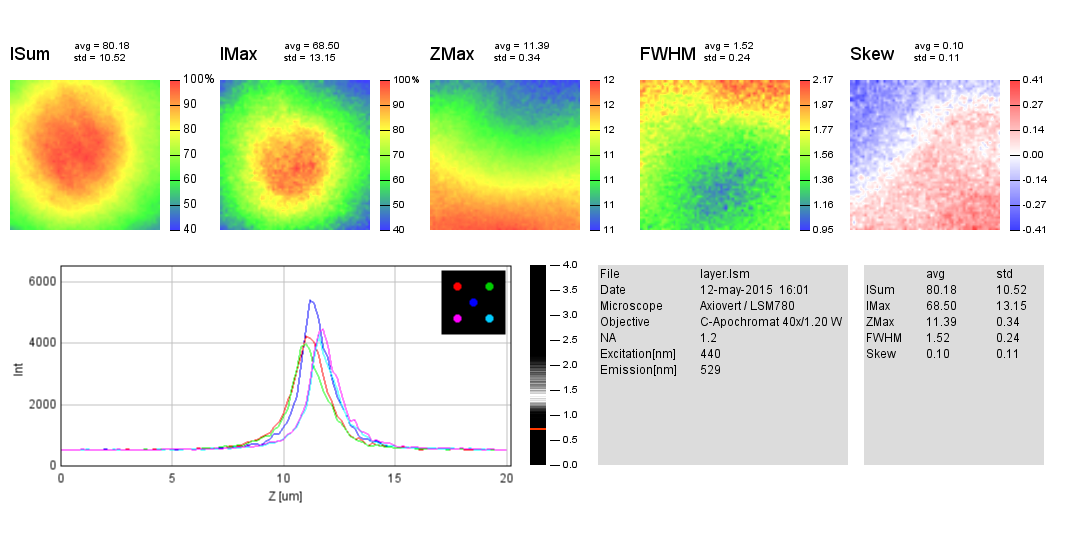

Supplement: S2 Fig — SIPchart generated with the ImageJ plugin from Norbert Vischer based on the SIPchart approach published by Brakenhoff et al. The data used for this analysis is identical with the layer data presented in Fig 2A. In general, the plugin produces comparable results but a reduced parameter set. The plugin utilizes a bin of 8x8 pixels reducing noise and spatial resolution. The intensity values are most likely derived from a conversion from 16 to 8 bit and reflect digital levels. (PNG) [file pone.0134980.s002.png]
