# Supplementary material for: eSIP: A Novel Solution-Based Sectioned Image Property Approach for Microscope Calibration
Source: PLoS One. 2015 Aug 5;10(8):e0134980. doi: 10.1371/journal.pone.0134980 (PMC4526552; doi:10.1371/journal.pone.0134980)
Supplement: S1 Table — To estimate the error of the fit parameters and to compare the eSIP fit approach with the SIPchart lookup approach we generated toy data by calculating gauss profiles (Eq 1) for layer and intensity profiles according to Eq 5 for solution. Maximal photon counts varying from 1 to 1000 photons and applying the corresponding Poisson noise and an additional Gaussian noise corresponding to the readout noise of the Zeiss LSM Quasar detector at detector gain 700 was applied. The following parameters have been used: data points layer (z), from -10 to +10 μm with 0.2 μm spacing; data points solution (z), from -5 to +5 μm with 0.1 μm spacing, from 6 to 50 μm with 1 μm spacing, from 55 to 200 μm with 5 μm spacing (same as in solution experiments); FWHM (ω FWHM), 1 μm; axial position (z 0), 0 μm; offset (I 0), 500 DL; photon conversion factor, 500 DL/photon, layer: skewness (s), -0.025, solution: length constant (LC), 0. We introduced an additional variation in the axial position (z 0) to avoid permanent coincidence of Gauss maximum and half-maximal values with pixel bins. In correlation to the settings in the presented work a 4x4 bin was used for analysis. Mean values and standard deviation (STD) were obtained from 500 simulations per intensity level. For the intensity (A) relative errors are provided as STD/mean. The error dependence with photon number is illustrated in S3 Fig. SIPchart lookup approach, layer: The Intensity (A) was found by searching for the brightest value. Since the z spacing is rather small compared to FWHM, A is notoriously too high at low photon counts. Contrary, for high photon counts it is too low. FWHM was determined by a line approximation between most outlaying data points above and the neighbouring data points below half maximal value. The axial position was found as the position of maximal intensity, so the accuracy directly correlates with z spacing. The offset value was estimated as average from ten values most distant from z0. The skewness was o [file pone.0134980.s005.pdf]

S1 Table. Toy data - Mean and standard deviation, obtained by the SIPchart lookup approach and the eSIP fit approach.

To estimate the error of the fit parameters and to compare the eSIP fit approach with the SIPchart lookup approach we generated toy data by calculating gauss profiles (Eq. 1) for layer and intensity profiles according to Eq. 5 for solution. Maximal photon counts varying from 1 to 1000 photons and applying the corresponding Poisson noise and an additional Gaussian noise corresponding to the readout noise of the Zeiss LSM Quasar detector at detector gain 700 was applied. The following parameters have been used: data points layer (z), from -10 to +10 µm with 0.2 µm spacing ; data points solution (z), from -5 to +5 µm with 0.1 µm spacing, from 6 to 50 µm with 1 µm spacing, from 55 to 200 µm with 5 µm spacing (same as in solution experiments); FWHM (ω_{FWHM}), 1 µm; axial position (z_0), 0 µm; offset (I_0), 500 DL; photon conversion factor, 500 DL/photon, layer: skewness (s), -0.025, solution: length constant (LC), 0. We introduced an additional variation in the axial position (z_0) to avoid permanent coincidence of Gauss maximum and half-maximal values with pixel bins. In correlation to the settings in the presented work a 4x4 bin was used for analysis. Mean values and standard deviation (STD) were obtained from 500 simulations per intensity level. For the intensity (A) relative errors are provided as STD/mean. The error dependence with photon number is illustrated in S3 Fig. **SIPchart lookup approach, layer:** The Intensity (A) was found by searching for the brightest value. Since the z spacing is rather small compared to FWHM, A is notoriously too high at low photon counts. Contrary, for high photon counts it is too low. FWHM was determined by a line approximation between most outlaying data points above and the neighbouring data points below half maximal value. The axial position was found as the position of maximal intensity, so the accuracy directly correlates with z spacing. The offset value was estimated as average from ten values most distant from z_0. The skewness was obtained (according to Zwier et al., 2006) as skew = (b-a)/(a+b) (compare S3 Fig F), which deviates from the eSIP approach definition Eq. 2 by a factor of about -2. **eSIP fit approach, layer:** Parameters were obtained by fitting Eq. 1 to the toy data. The look up parameter were used as start parameters for the fit. As expected, an improvement of the accuracy and lower standard deviations of the fit parameters were obtained. **eSIP fit approach, solution:** Parameters were obtained by fitting Eq. 5 to the toy data. The length constant LC was not fitted. The accuracy as deviation of the mean from the input parameter and the precision in terms of STD are illustrated in S3 Fig. Unless toy data are only provided for one most relevant parameter set, this demonstrates the quality of all approaches, whereas the eSIP fit approach for solution reaches at least results of the same quality as the SIPchart approach for layer.

SIPchart look up approach, layer					
Intensity input [Photon]	Intensity (A) [Photon]	FWHM (ω_{FWHM}) [µm] (input: 1 µm)	Axial position (z_0) [µm] (input: 0 µm)	Offset (I_0) [DL] (input: 500 DL)	Skewness (skew) (input: 0.0125*)
1.00	1.14 (±16.78 %)	0.91 ± 0.207	0.001 ± 0.183	499.8 ± 8.701	0.0242 ± 0.393
1.26	1.43 (±15.21 %)	0.90 ± 0.191	-0.004 ± 0.161	500.3 ± 8.299	0.0236 ± 0.364
1.58	1.77 (±13.92 %)	0.91 ± 0.166	-0.009 ± 0.158	499.8 ± 8.126	0.0378 ± 0.344
2.00	2.18 (±13.42 %)	0.93 ± 0.149	-0.002 ± 0.153	499.8 ± 8.076	0.0141 ± 0.334
2.51	2.69 (±12.25 %)	0.95 ± 0.136	-0.005 ± 0.145	499.7 ± 8.205	0.0207 ± 0.313
3.16	3.35 (±10.19 %)	0.96 ± 0.126	-0.012 ± 0.131	500.1 ± 8.272	0.0339 ± 0.291
3.98	4.19 (± 9.63 %)	0.96 ± 0.108	0.004 ± 0.129	500.1 ± 8.454	0.0051 ± 0.280
5.01	5.17 (± 8.38 %)	0.98 ± 0.090	-0.002 ± 0.124	500.4 ± 8.308	0.0113 ± 0.262
6.31	6.48 (± 7.30 %)	0.98 ± 0.086	0.000 ± 0.117	499.7 ± 8.015	0.0161 ± 0.246
7.94	8.10 (± 7.37 %)	0.99 ± 0.080	-0.002 ± 0.108	499.4 ± 8.138	0.0157 ± 0.224
10.00	10.23 (± 6.39 %)	0.98 ± 0.070	-0.002 ± 0.101	499.7 ± 8.625	0.0221 ± 0.207
12.59	12.75 (± 5.51 %)	0.99 ± 0.060	0.002 ± 0.097	500.1 ± 8.570	0.0072 ± 0.198
15.85	15.99 (± 5.22 %)	1.00 ± 0.055	0.005 ± 0.090	500.8 ± 8.246	0.0013 ± 0.185
19.95	19.99 (± 4.71 %)	1.00 ± 0.050	-0.005 ± 0.088	500.0 ± 8.280	0.0241 ± 0.178
25.12	25.20 (± 4.40 %)	1.00 ± 0.045	0.007 ± 0.086	500.2 ± 8.161	0.0019 ± 0.175
31.62	31.45 (± 3.75 %)	1.01 ± 0.038	0.002 ± 0.083	500.0 ± 8.132	0.0080 ± 0.168
39.81	39.66 (± 3.45 %)	1.01 ± 0.035	-0.000 ± 0.077	499.4 ± 8.272	0.0135 ± 0.155
50.12	50.00 (± 3.16 %)	1.00 ± 0.031	0.003 ± 0.075	500.7 ± 8.371	0.0095 ± 0.152
63.10	62.94 (± 2.82 %)	1.01 ± 0.029	0.004 ± 0.071	500.0 ± 8.005	0.0050 ± 0.141
79.43	79.11 (± 2.63 %)	1.01 ± 0.027	0.001 ± 0.071	499.5 ± 8.521	0.0108 ± 0.141
100.00	99.29 (± 2.38 %)	1.01 ± 0.023	-0.003 ± 0.064	500.0 ± 8.586	0.0185 ± 0.128
125.89	125.14 (± 2.12 %)	1.01 ± 0.021	0.001 ± 0.063	499.7 ± 8.460	0.0115 ± 0.124
158.49	157.13 (± 1.95 %)	1.01 ± 0.019	-0.003 ± 0.064	499.9 ± 8.019	0.0202 ± 0.126
199.53	197.92 (± 1.79 %)	1.01 ± 0.017	0.006 ± 0.063	500.0 ± 8.206	0.0007 ± 0.125
251.19	248.96 (± 1.69 %)	1.01 ± 0.015	-0.003 ± 0.064	499.6 ± 8.583	0.0193 ± 0.125
316.23	313.63 (± 1.59 %)	1.01 ± 0.014	0.000 ± 0.061	500.0 ± 7.875	0.0121 ± 0.118
398.11	394.77 (± 1.47 %)	1.01 ± 0.013	-0.003 ± 0.061	500.2 ± 8.161	0.0176 ± 0.118
501.19	497.08 (± 1.34 %)	1.01 ± 0.011	-0.002 ± 0.060	500.1 ± 8.071	0.0160 ± 0.119
630.96	625.35 (± 1.19 %)	1.01 ± 0.010	-0.003 ± 0.059	500.3 ± 7.979	0.0183 ± 0.115
794.33	787.33 (± 1.13 %)	1.01 ± 0.009	0.002 ± 0.056	499.8 ± 8.113	0.0083 ± 0.109
1000.00	991.74 (± 1.05 %)	1.01 ± 0.008	0.002 ± 0.056	500.7 ± 8.281	0.0100 ± 0.110

eSIP fit approach					
Intensity input [Photon]	Intensity fit (A) [Photon]	FWHM (ω_{FWHM}) [µm] (input: 1 µm)	Axial position (z_0) [µm] (input: 0 µm)	Offset (I_0) [DL] (input: 500 DL)	Skewness (s) (input: -0.025)
1.00	0.98 (±16.27 %)	0.93 ± 0.113	0.011 ± 0.089	499.8 ± 2.655	-0.0051 ± 0.154
1.26	1.24 (±14.15 %)	0.93 ± 0.098	0.000 ± 0.078	499.9 ± 2.475	-0.0269 ± 0.138
1.58	1.58 (±12.19 %)	0.94 ± 0.083	0.003 ± 0.068	499.7 ± 2.607	-0.0170 ± 0.122
2.00	2.01 (±11.34 %)	0.94 ± 0.077	0.000 ± 0.060	500.3 ± 2.758	-0.0176 ± 0.112
2.51	2.53 (± 9.41 %)	0.95 ± 0.060	0.001 ± 0.051	499.7 ± 2.700	-0.0207 ± 0.096
3.16	3.19 (± 7.93 %)	0.96 ± 0.055	-0.003 ± 0.045	499.9 ± 2.653	-0.0274 ± 0.084
3.98	4.01 (± 7.16 %)	0.97 ± 0.049	0.001 ± 0.041	499.8 ± 2.934	-0.0248 ± 0.074
5.01	5.04 (± 6.31 %)	0.97 ± 0.041	-0.002 ± 0.036	500.1 ± 2.951	-0.0243 ± 0.061
6.31	6.36 (± 5.29 %)	0.97 ± 0.036	0.002 ± 0.032	499.8 ± 2.728	-0.0200 ± 0.054
7.94	8.00 (± 4.97 %)	0.98 ± 0.034	0.002 ± 0.028	499.8 ± 2.760	-0.0242 ± 0.049
10.00	10.08 (± 4.18 %)	0.98 ± 0.029	0.002 ± 0.026	499.5 ± 2.966	-0.0241 ± 0.043
12.59	12.70 (± 3.96 %)	0.98 ± 0.026	0.000 ± 0.022	499.5 ± 2.760	-0.0239 ± 0.038
15.85	15.96 (± 3.44 %)	0.98 ± 0.022	0.000 ± 0.019	499.9 ± 2.815	-0.0246 ± 0.033
19.95	20.03 (± 3.11 %)	0.99 ± 0.021	0.002 ± 0.017	499.8 ± 2.982	-0.0238 ± 0.029
25.12	25.32 (± 2.80 %)	0.99 ± 0.018	0.002 ± 0.015	499.9 ± 2.902	-0.0233 ± 0.025
31.62	31.70 (± 2.43 %)	0.99 ± 0.016	-0.000 ± 0.014	499.5 ± 2.852	-0.0245 ± 0.023
39.81	39.99 (± 2.09 %)	0.99 ± 0.014	0.000 ± 0.012	499.5 ± 2.755	-0.0242 ± 0.020
50.12	50.30 (± 1.99 %)	0.99 ± 0.013	0.001 ± 0.011	499.9 ± 2.815	-0.0243 ± 0.018
63.10	63.37 (± 1.65 %)	0.99 ± 0.010	0.001 ± 0.010	499.4 ± 2.764	-0.0239 ± 0.015
79.43	79.78 (± 1.45 %)	0.99 ± 0.010	0.000 ± 0.009	499.8 ± 2.810	-0.0250 ± 0.014
100.00	100.32 (± 1.35 %)	1.00 ± 0.008	-0.000 ± 0.008	499.7 ± 2.944	-0.0252 ± 0.012
125.89	126.24 (± 1.16 %)	1.00 ± 0.008	0.001 ± 0.007	499.6 ± 2.886	-0.0246 ± 0.012
158.49	158.82 (± 1.12 %)	1.00 ± 0.007	0.001 ± 0.006	499.5 ± 2.861	-0.0243 ± 0.010
199.53	199.92 (± 0.95 %)	1.00 ± 0.006	0.000 ± 0.005	499.4 ± 2.982	-0.0244 ± 0.009
251.19	251.53 (± 0.86 %)	1.00 ± 0.005	0.001 ± 0.005	499.5 ± 3.102	-0.0239 ± 0.007
316.23	316.59 (± 0.76 %)	1.00 ± 0.005	0.000 ± 0.004	499.5 ± 2.865	-0.0247 ± 0.007
398.11	398.61 (± 0.64 %)	1.00 ± 0.004	0.000 ± 0.004	499.6 ± 2.889	-0.0244 ± 0.006
501.19	501.77 (± 0.60 %)	1.00 ± 0.003	0.000 ± 0.003	499.3 ± 2.852	-0.0248 ± 0.005
630.96	631.33 (± 0.50 %)	1.00 ± 0.003	-0.000 ± 0.003	499.4 ± 2.772	-0.0251 ± 0.005
794.33	794.73 (± 0.44 %)	1.00 ± 0.003	0.000 ± 0.003	499.7 ± 2.745	-0.0249 ± 0.004
1000.00	1000.81 (± 0.43 %)	1.00 ± 0.003	0.000 ± 0.002	499.6 ± 2.968	-0.0245 ± 0.004

eSIP fit approach Solution				
Intensity input [Photon]	Intensity fit (A) [Photon]	FWHM (ω_{FWHM}) [µm] (input: 1 µm)	Axial position (z_0) [µm] (input: 0 µm)	Offset (I_0) [DL] (input: 500 DL)
1.00	0.91 (± 3.58 %)	0.93 ± 0.267	0.043 ± 0.110	500.6 ± 4.193
1.26	1.18 (± 2.66 %)	0.93 ± 0.218	0.038 ± 0.085	500.4 ± 4.173
1.58	1.51 (± 2.21 %)	0.93 ± 0.183	0.033 ± 0.071	500.1 ± 4.397
2.00	1.93 (± 1.90 %)	0.94 ± 0.156	0.028 ± 0.063	500.3 ± 3.949
2.51	2.44 (± 1.65 %)	0.94 ± 0.128	0.018 ± 0.051	500.3 ± 4.243
3.16	3.09 (± 1.42 %)	0.93 ± 0.114	0.015 ± 0.045	500.1 ± 4.073
3.98	3.91 (± 1.23 %)	0.94 ± 0.101	0.012 ± 0.039	500.2 ± 4.200
5.01	4.95 (± 1.03 %)	0.95 ± 0.084	0.007 ± 0.034	499.9 ± 4.177
6.31	6.24 (± 0.97 %)	0.96 ± 0.077	0.009 ± 0.030	500.2 ± 4.109
7.94	7.88 (± 0.86 %)	0.96 ± 0.070	0.004 ± 0.026	499.5 ± 4.335
10.00	9.93 (± 0.70 %)	0.96 ± 0.058	0.004 ± 0.023	499.8 ± 4.366
12.59	12.53 (± 0.64 %)	0.96 ± 0.052	0.001 ± 0.020	499.6 ± 4.440
15.85	15.79 (± 0.58 %)	0.97 ± 0.044	0.001 ± 0.018	499.9 ± 4.274
19.95	19.90 (± 0.53 %)	0.97 ± 0.041	0.000 ± 0.016	499.7 ± 4.069
25.12	25.05 (± 0.46 %)	0.97 ± 0.034	-0.000 ± 0.013	499.5 ± 4.356
31.62	31.58 (± 0.45 %)	0.98 ± 0.031	-0.000 ± 0.012	499.8 ± 4.475
39.81	39.73 (± 0.35 %)	0.98 ± 0.028	-0.000 ± 0.011	499.5 ± 4.501
50.12	50.04 (± 0.33 %)	0.99 ± 0.024	-0.001 ± 0.010	499.8 ± 4.423
63.10	63.01 (± 0.27 %)	0.99 ± 0.022	-0.001 ± 0.009	499.6 ± 4.661
79.43	79.36 (± 0.26 %)	0.99 ± 0.019	-0.000 ± 0.008	499.4 ± 4.182
100.00	99.92 (± 0.24 %)	0.99 ± 0.017	-0.001 ± 0.007	499.4 ± 4.410
125.89	125.81 (± 0.22 %)	0.99 ± 0.016	-0.001 ± 0.006	499.5 ± 4.697
158.49	158.38 (± 0.18 %)	0.99 ± 0.013	-0.000 ± 0.005	499.7 ± 4.483
199.53	199.42 (± 0.16 %)	0.99 ± 0.012	-0.001 ± 0.005	499.3 ± 4.427
251.19	251.08 (± 0.15 %)	0.99 ± 0.009	-0.001 ± 0.004	499.0 ± 4.662
316.23	316.14 (± 0.13 %)	1.00 ± 0.009	-0.001 ± 0.004	499.1 ± 4.644
398.11	398.03 (± 0.12 %)	1.00 ± 0.008	-0.001 ± 0.004	498.9 ± 4.824
501.19	501.15 (± 0.10 %)	1.00 ± 0.007	-0.000 ± 0.003	499.0 ± 4.597
630.96	630.86 (± 0.09 %)	1.00 ± 0.006	-0.000 ± 0.003	498.8 ± 4.732
794.33	794.26 (± 0.08 %)	1.00 ± 0.006	-0.000 ± 0.003	498.8 ± 4.654
1000.00	999.89 (± 0.07 %)	1.00 ± 0.005	-0.000 ± 0.002	499.1 ± 4.886
